# Hypnotism, Animal Magnetism, and Personal Magnetism as Applied to Dentistry

**Published:** 1894-01

**Authors:** W. G. A. Bonwill


### HYPNOTISM, ANIMAL MAGNETISM, AND PERSONAL MAGNETISM AS APPLIED TO DENTISTRY.[1]

BY W. G. A. BONWILL, D.D.S.

Mr. President,—What do I know about hypnotism, you might well ask?

Does any one know anything definite as to the truth of it; or, if genuine, who can explain how it is done, or can it be reduced to a science to be depended upon?

The object of this paper is to show that hypnotism plays only a small percentage as an obtunder of pain; that hypnotism is due to a certain condition of the subject and not to the power of the one operating; that there are various means to produce temporary effects favorable as helps; that in analgesia we have a condition of suspension of the voluntary so far as to increase sense of touch and sound, and yet to completely annul pain, and that it can be produced in many ways; that it is not suggestion but strong impression made by the operator; the deaf can be influenced through their language, but it must be impressive; that it is not necessary in any case to try whether the patient is in a natural hypnotic state or that this can be induced by artifical means.

When once the operator will study his case and follow directions here given, success will come, as the will or voluntary brain can be suspended and held in abeyance. Not every man can command or govern. Some men can and not fail in anything they undertake.

As horses are managed by making them understand there is a master, so with human beings.

Its history is so well known that it is not necessary to make more than a brief allusion to it. Like all other good things, it has had a most terrific fight for its existence. Many brave men have gone down because they asserted it was a fact; many reputations have been forever blasted for advocating it. And to-day it is far from being believed by scientific men, although many have espoused its cause, and the learned societies are gradually endorsing it as a great principle, while its action is not in any way understood. For half a century a war has been waged against it, and still it lives, and it will endure.

As early as 1733, Mesmer, a German physician, first propounded

---

[1] Read before the Odontological Society of Pennsylvania, November 11, 1893.

the theory. It was then known as Mesmerism, animal magnetism, psychology, and personal magnetism, but now more scientifically named hypnotism, or artificial somnambulism. It was at one time called Braidism, after an English surgeon, who was the first to prosecute its phenomena scientifically. Mesmer claimed the effect was produced by the power of magnets in the hands of an expert. It was claimed that the will of the operator could control that of another by a certain animal magnetism going out of his (the operator's) body, and only a few could claim such power. It is now held that no force of any kind emanates from the operator. It is regarded by many as artificial catalepsy.

Hypnotism more fully expresses the meaning, "putting to sleep." It is also an induced somnambulism. That there is a certain percentage of persons who are susceptible to it is a fact. Why they are so no one, so far, can even surmise.

It was opposed by Christians because of its miraculous effects, they believing the day had passed for such wonders. The Catholic church held that it alone had such power. Scientific men opposed it on the ground that what could not be scientifically or philosophically explained could not be true, and because they could not see the *modus operandi* they refused it a hearing.

It is not surprising that it should have had such opposition, for it claimed much, and its effects were marvellous. I remember when I first witnessed the experiments I was quite a boy; it excited my curiosity, and struck terror to me.

In 1855 a class was formed to learn its mysteries. We each paid five dollars for the instruction, with a written pledge that we would not reveal the secret. We were allowed to practise it privately or publicly, but must hold the pledge inviolate.

That five dollars was the best investment I ever made, for it opened my eyes to what might never have been revealed to me.

While much has been taught and demonstrated, yet there remains much to be revealed to enable the majority of men to do what seemed impossible to them. Dr. Fillebrown has written for the benefit of dentists, but he does not give up the key to its action, as I conceive should be done, to enable others to practice it. He refers you to Bernheim. The common belief that only weak-minded persons were good subjects has been exploded.

It is certain that those of strong resistance or strong controlling will power cannot be good subjects. Those who have little resistive power can become good subjects. Fifty per cent. of adults are susceptible; most children can be directed; more women than men. The possession of the power has led to many abuses, and it is well to be on guard for unscrupulous men.

It is a means by which one can readily produce in the mind of the subject such a lasting impression as to injure health, or it may promote it.

The action is not, *per se*, abnormal, yet in the play upon the imagination can leave the subject a prey to the suggestion or command of the operator.

That it is the means of curing diseases both organically and functionally, when intelligently used, should be believed. Physicians who have relied solely upon medicine for therapeutic effects should acquaint themselves with its value and no longer saturate their patients with poisonous doses that do more harm than good.

Much of the prejudice of the public would be overcome if they were properly taught that it is not so much in one person being able to hold another in subjection as that they, or a certain percentage, are liable to be controlled simply from a peculiar condition of their own body and mind.

While it takes several minutes before any change takes place to enable one to control and direct thought and action, in many cases but a very brief period will suffice. A command energetically made by one having the gift of ordering another to obey will be sufficient, and it is not necessary that it should be acted on at the instant, as the impression can be left on the mind, and in days after the command will positively be obeyed.

When we survey the subject in the phases of every day existence we find our actions are governed by influences we cannot fathom. We know we are led captive by the power of woman, and, *vice versa*, women by men. The presence of an animal will paralyze persons and make them lose all control of their judgment. We listen to an orator and are led captive not so much by his reasoning, but by his presence, his voice, his every gesture. There evidently is some force going out from one who has personal magnetism, although it may not be the same as hypnotism. While but few are born with such power, it can be cultivated and practised.

You hear of induction in electricity. It is as wonderful as hypnotism. There you see actual power manifested through the medium of the air, and it is only a mechanical force. Is it to be doubted that man possesses a power that may pass from him and affect another and force him to obey commands?

You tell a person to meet you at a certain time. The voice is the medium of communication, and the vibration is manifested as would be induction from the vibrations of a dynamo.

An hypnotic patient or subject becomes an automaton, and will run as a machine, without any power to resist. That the human mind can be dominated by an idea alone, without it coming as a command from another is often seen in cases of crime. In natural sleep the mind can become somnambulistic, and such subjects act as if they were perfectly awake and in their right mind.

Faith has done much to remove actual organic diseases. Christian Science has accomplished much in its peculiar way to rid sufferers of bodily and mental ailments through teaching self-control and a belief in their own powers. The history of the human race gives thousands of facts as to cures from various methods, long before hypnotism or the assumed power of magnets were conceived and practised. Observe how many people wear magnetic rings upon a certain finger, positive as to their magic therapeutic effects. It matters not under what name the treatment is made, certain effects and cures are produced. The various modes of medical practice, as extreme and antipodal as possible, yet, each and all, do a work not to be gainsaid.

So much has been demonstrated through investigations upon this subject that gradually the real cause of all such phenomena has been brought to light, and it would seem that we are upon the threshold of an actuality which may dispel the darkness that veiled the hidden mysteries to men.

It was not until M. Leibault, in 1862, after continued experiments with hypnotism in the hospital at Nancy, in France, was it taken up by the better class of medical and scientific men and received extended acknowledgment. His therapeutic applications were so satisfactory that the power could be relied upon in the many and various cases he relates in his exhaustive work on "Hypnotic Suggestion." To him should be given the credit for the absolute system of treatment through this agent.

Then it is to "suggestion" alone, as he has named it, that we are to rely for our success. But you are not to understand that the merely asserting to your subject that a certain effect can and will be produced upon him will accomplish the object, for there is a condition of mind and body that your patient must already have or gain, without which suggestion will fail.

When I was taught animal magnetism, or psychology, as then known, in 1855, it led me to adopt it partially, and it was modified to suit my practice. It is one of many adjuncts in gaining the confidence of my patients and placing them more completely under my control in causing my operations to be less painful and giving wonderful assistance in making my practice more satisfactory to my patient and myself.

I found there was such a thing as personal magnetism, which has no relation to hypnotism. I am sure that some men can have such a personal force that they can control others and draw to them, and, while it can be cultivated to a considerable degree in many, it is a born quality or power by which good or evil may be done.

Some persons can be overcharged with electricity, both frictional and from dynamic source, enabling them to light the gas. Is it any more to be doubted they can have other powers? When I first promulgated rapid breathing I was charged with being dishonest, as it was believed I performed this through personal magnetism.

I have assured you that it is a fact not to be ignored that hypnotism is all that is claimed for it, both by empirics and by some scientific men, in its action upon the normal subject and in its therapeutic powers.

The history of hypnotism informs us that from the very first it has been used, not for amusement only, but as a great healing agent, and so wonderful were the results that scientific men have been compelled to bow to the inevitable.

In commencing to operate, the command must be given as a general of an army would issue an order. It is in the way it is done. For instance, if I were to simply go up to my subject and whisper in a low tone without any positive force, he would not be impressed with the order and most likely not comply. But when I say to him, in a loud, stentorian voice, that he *cannot do so and so*, or he *must do thus and so*, he has no will to do other than I command.

Now, this is a peculiarity of hypnotic subjects. It is different, as I think I told you, with personal magnetism. Forty per cent. are subject to this hypnotic state, or can soon be placed so by the simple concentration of their minds upon any one thing, as my teacher did by simply having the individual close the eyes and count his pulse for fifteen minutes.

If you will pause just here you will see that it is the key to the philosophy of sleep.

I took the hint from this, and when inclined to insomnia I would place myself in bed in some easy position, and with one finger—not all my fingers—would commence to scratch my head very gently. My attention then was riveted on what I was doing. While thus engaged my mind was directed to this alone, and it could not, so long as I kept up this gentle scratching, let it run on a second thought. If you accept this you see how easy it is for sleep to take place, for a few minutes at least when the mind is subjugated to the tyranny of *one* thought. It is dominated by one idea. You will find that the same thing is not attained if you use all the fingers of the one hand. It gives the brain too much to do, and is rather an excitant than a soporific. The same effect is produced by rapid breathing for a minute. The mind is diverted for that time, and with the rapid accumulation of carbonic oxide gas in the circulation, a double effect is produced.

Do you not grasp, then, that if it is impossible for any one to sleep with a thousand thoughts flying through their brain, that if the mind can be brought down to one thing it becomes a blank as far as other thoughts are concerned?

Take away your volition or power to control your voluntary actions, and you become an automaton, and are a prey to sleep because all is a blank, and only the involuntary life goes on.

The two principles in our system, the voluntary and involuntary, are acting entirely independent of each other, and to produce sleep the voluntary must be suspended, and this is secured when you take the step to draw upon your voluntary to keep your finger going.

Do you suppose that the performer upon a piano could for a moment go to sleep with all his fingers flying? The action forbids it. The brain is under too great a strain, and until complete exhaustion is produced sleep cannot come.

When you stop to reason or philosophize you see how much more rational hypnotism seems. It is no longer a mystery, but becomes a part of sleep itself.

You have another proof of profound sleep in the use of ether, chloroform, or any of the anæsthetics. It is seen in morphia and all such preparations. It is simply the annulling for the time being all voluntary life, and hence volition is no longer dominant,—the machine proper is running itself without any governor.

Another factor, not less potential, is "dead silence." Did you ever try to keep "still" for five minutes? Try it, and you have an effect of the voluntary subduing itself. Try to keep from moving a finger or blinking, or, if your eyes are open, try not to close them. The effect will be to bring that perfect repose the nearest kin to sleep.

There is yet another factor in this wonderful machinery of nature. From my experiments in rapid breathing I discovered that natural sleep will always result from legitimate labor during the daylight, when the heart and lungs have been over active, while the body is under action in all its parts. The excessive amount of oxygen taken while laboring sets free carbonic oxide gas more freely, and it passes to the lungs to escape.

At night, when the body and, of a sequence, all its organs are powerless to act at a normal speed, particularly the heart and lungs, the carbonic oxide accumulates in the tissues faster than it can be carried off by the sluggish heart and less active lungs, as the vehicle to convey it out of the system; and hence the effect of the carbonic oxide is the same as if you had actually breathed it in from the outside air.

Now, you should know that this poisonous gas has this soporific effect, and would extinguish the flame of life as well as it will that of a candle, if inhaled for a short period. But in this normal inaction of the heart and lungs sleep is most beautifully courted.

The rapid movements of the body during the day in taking in the excess of oxygen sets free from this normal activity of the function all the carbonic oxide gas as fast as the oxygen grasps it in its movements through the circulation. But in this case the heart and lungs are acting in harmony, as one to four. As soon as the day's labor is over the heart, while making its normal number of beats, is less forcible, and its capacity is not so great, while the lungs are less active to meet the demands of a weakened heart, and, at the same time, the involuntary action of every organ in the body goes on in order that the exhausted body, from the day's labors, shall be supplied with nutrition before the dawn of another day, and all the organs shall be renewed with vigor; and, while all this is progressing, the carbonic oxide gas is not thrown to the lungs to escape, and it has its effect upon the brain while it is lingering in the tissues and nerve-centres and the central organ the brain. Is not this a rational conclusion?

If you will argue this out as I have done you must accept the conclusion of the philosophy of sleep, and that the carbonic acid gas is the active agent in its promotion; and that hypnotism, or, in other words, artificial sleep, has carbonic acid gas as one of its principal factors.

I will now take you a step farther. You understand that the principal thing to be considered in hypnotism is what is now called "suggestion." That is, the hypnotic subject would always remain himself unless some one were to step up and command or suggest or order him to do something against his own will, which may be now at its lowest ebb. You must place some thought before his mental vision, or through his sense of hearing alone, and with force demand its recognition, which is more than a mere suggestion.

This demand must be kept up or a series of thoughts put before his mind which will lead him to carry on a system of action.

While this must be done to be in any way successful in hypnotism, it can be used in every-day practice of dentistry and medicine without the patient being hypnotized.

It is astonishing how nearly every patient can be effected by forcibly declaring to them, "It will not hurt you." "If I am going to hurt you I will tell you." "Fear not, for I can assure you that you will be quite or entirely free from pain."

Children are easily controlled if their attention can be secured. But they are addicted to spasmodic crying, and until that is over it is impossible to do much with them.

Finally, I will lead you a step farther to the mode adopted to produce effects on patients which, while not hypnotic, enables me to do whatever I please to their teeth.

*First Stage.*—When the door bell is rung to have my maid go as quickly as possible and never keep the subject waiting in suspense for a moment. The maid is bidden to always show a pleasant face and to be very polite. Patient is ushered into the hall and a beautiful Madonna greets the eye. A step on and a large mirror that covers the hall to the ceiling gives the hallucination that the house has a very long hall and a series of rooms. This effect is produced by another large mirror directly opposite in the parlor. The parlor should be open, and while not extravagantly furnished, should present a pleasant view. The view, as one proceeds up the steps, should be to attract attention, and, the drawing-room gained, the maid gives any attention needed or demanded. Books or papers are given them instead of letting them sit in idleness until the operator presents himself.

As soon as possible he should go to the patient and speak in such manner and grasp the hand with a touch that inspires confidence. If a stranger, take one minute to give them something pleasant to look at or read. Handsome engravings are always entertaining to young and old. Have plenty of chairs, that a choice can be made. Have nothing so fine and luxurious in this drawing-room that patients are afraid to touch or disarrange. Let everything be free and easy. Assure them you will keep them waiting but a moment. If extremely nervous, then I have them sit profoundly still, and draw their attention to one object that this hypnotic effect may be cultivated or drawn out, if they are possessed of it, that they will be very much better prepared for the operator. Never let patients tell you a word about their case. Direct them to keep still or silent, and not even hint where the trouble lies. Find it yourself, and you have gained their confidence at once. Have them wait, and allow no one to leave your office until you have done something to soothe their pain and allay their fears. Assure them that they are not so badly off as they surmised. Do not fear to spend a moment in conversation to them on leaving, and forcibly and smilingly assure them not to think of you while away and up to the time of next visit. Do not let your maid attend to this part of your office. Nothing can take your place.

So much for the entering wedge—the *entrée* into your house and office—to have an hypnotic effect.

While your voice is the principal thing over all to bring out the result, these minor points speak volumes in silence to captivate and ensnare your subject.

Some I would have sit with their finger on the pulse for ten or fifteen minutes before I commence examination. To a thoughtful operator there are many avenues by which he can hypnotize,—electrically by induction, personal magnetism, etc. All of this and more I could give you.

Now, while I have written this essay on hypnotism, and have shown you that it is a great fact, and have attempted its solution, yet I must impress upon you to speak with decision, and never aim to discover in advance whether the subject is an hypnotic.

To one conversant with personal magnetism, the fact can be relied upon, that in the voice, look, and touch, the bulk of people can be ruled or controlled, and no hypnotism about it. By diverting the attention or mind at the instant, there is no failure where the pain is instantaneous. When extracting temporary teeth this is all I do, and before the little patient can take one long breath the tooth or teeth are removed.

There are many cases where I produce a more decided effect by playing on the imagination, by having them breathe the air through a tube, as if an anæsthetic were being given. Some minds are very susceptible to such a method. Do not fail to accept the fact that, aside from all these little subterfuges, without saying a word, while breathing I can universally affect them.

In conclusion, cultivate the sense of touch, sight, and voice to a great degree, and be always truthful, and, with sharp instruments, used with skill and great rapidity, you will not hurt them; you have in your grasp the many means by which dentistry may be robbed of all its horrors.

